# Sex Differences in the Acute Effects of Early Partial and Total Sleep Deprivation on Strength, Power, and Endurance Performance in Resistance-Trained Participants

**DOI:** 10.3390/jfmk11010083

**Published:** 2026-02-19

**Authors:** Marta del Val-Manzano, Juan Jesús Montalvo-Alonso, Paola Gonzalo-Encabo, David Valadés, Carmen Ferragut, Alberto Pérez-López

**Affiliations:** Departamento de Ciencias Biomédicas, Área de Educación Física y Deportiva, Facultad de Medicina y Ciencias de la Salud, Universidad de Alcalá, 28805 Madrid, Spain; marta.val@uah.es (M.d.V.-M.); jesus.montalvo@uah.es (J.J.M.-A.); paola.gonzalo@uah.es (P.G.-E.); david.valades@uah.es (D.V.); carmen.ferragut@uah.es (C.F.)

**Keywords:** sex characteristics, sleep, sleep deprivation, muscle strength, muscle fatigue, resistance exercise

## Abstract

**Background**: Sleep is essential for athletic performance, yet the specific effects of sleep deprivation are not well defined. Evidence in resistance-trained populations is limited regarding sex-specific responses and velocity-based performance across different loads. **Purpose:** This study examined sex differences in the impact of total (0 h) and partial (4 h) sleep deprivation versus normal sleep (8 h) on strength, power, and endurance performance in resistance-trained individuals. **Methods**: Twenty-four resistance-trained participants (male/female, 12/12; age: 22 ± 3 years) completed a randomized, cross-over, counterbalanced trial including one baseline control night (8 h at home sleep) and three experimental conditions in the laboratory: (a) 8 h sleep (NS), (b) 4 h sleep (ESD), (c) 0 h sleep (SD). Strength was assessed at 25%, 50%, 75%, 90% and 100% 1RM for bench press and back squat (half-squat depth, ~90° knee flexion), in a Smith machine, followed by a muscular endurance test at 65% 1RM (set-to-failure). Isometric strength and vertical jump test were also performed. **Results**: At 50% 1RM, significant sleep and sleep-by-sex effects were observed for V_mean_ in both exercise (*p* < 0.05, η_p_^2^ > 0.09), an effect only noted in males, with reduced performance under ESD and SD compared to NS (7–13%, *p* < 0.05, *g* > 0.50). In the muscular endurance test, sleep and sleep-by-sex effects were found (*p* < 0.05, η_p_^2^ < 0.22), an effect only found in females during the back squat, showing performance declines in V_mean_ in ESD and SD compared to NS (7–12%, *p* < 0.05, *g* > 0.2). **Conclusions**: Total and partial sleep deprivation impairs muscular performance differently by sex. Males experienced reduced strength at moderate loads, while females showed declines in muscular endurance.

## 1. Introduction

Sleep is a key factor in athletic performance, supporting recovery, cognition, and physical capacity [1,2]. The effects of partial and total sleep deprivation on muscular strength, power, and endurance are complex and vary among individuals [3,4,5]. Evidence also suggests biological sex may influence these effects [6,7], though this remains underexplored, especially in resistance-trained populations.

Sleep restores immune, endocrine, central nervous, and metabolic functions [8,9,10]. Adults are advised to obtain 7–9 h per night [11], yet many fail to do so [12]. Sleep deprivation—including early sleep deprivation—is defined as a reduction or restriction of sleep during the initial part of the night; it can disrupt normal sleep architecture (i.e., the pattern and proportion of non-rapid eye movement (NREM) and rapid eye movement (REM) sleep stages across the night), which is critical for physiological recovery and neuromuscular restoration. Athletes face a heightened risk due to the demands of training and competition [13]. Insufficient sleep reduces well-being [14], productivity (e.g., work absence and presenteeism) [15], and safety (e.g., the ability and capacity to respond and compensate for changed internal and external environments) [16], and in athletes, it impairs physiology and performance by lowering muscular strength (e.g., maximal weight lifted in bench press, leg press and dead lift exercises) [5] and endurance (e.g., reduced speed in a 3 km cycling time-trial performance) [17], and cognitive-motor function [18], while increasing perceived effort [19].

Over recent decades, systematic reviews and meta-analyses have shown that sleep loss generally impairs athletic performance, including reductions in muscular strength and endurance; however, these studies primarily employed isometric or isokinetic assessments and involved predominantly male participants (~75%) [20]. More recent evidence has expanded this understanding by clarifying the mechanisms and moderators involved. A systematic review by Easow et al. [21] reported that both acute (i.e., one or a few nights of markedly reduced sleep) and chronic (i.e., repeated or prolonged partial sleep loss across multiple nights) sleep restriction reduce muscular strength, power, and endurance, alongside impaired neuromuscular function and increased fatigue. Similarly, comparisons between athletes and non-athletes show that sleep deprivation significantly impairs aerobic endurance, maximal force, movement skill, and speed, increases perceived exertion, and produces greater performance decrements during afternoon testing [22]. Of note, only 3 of the 52 studies included in this systematic review and meta-analysis recruited a sex-balanced sample. Additionally, a review of sleep interventions in athletes found that sleep extension and daytime napping effectively mitigate performance declines associated with insufficient sleep, highlighting the practical importance of sleep management in sport [23]. Despite these advances, evidence regarding sex differences remains limited. Recent observational studies suggest that female athletes may experience more frequent sleep disturbances and a higher prevalence of sleep medication use compared to males, indicating potential sex-related vulnerabilities or differing sleep–performance relationships [24,25]. However, prior research in resistance-trained populations has not examined dynamic, velocity-based performance outcomes across a broad spectrum of relative loads (25–100% 1RM), nor has it evaluated sex-disaggregated responses to both partial and total sleep deprivation within the same crossover protocol. This critical gap underpins the present study, which investigates sex-specific effects of total (0 h) and partial (4 h) sleep deprivation on strength, power, and endurance performance in resistance-trained male and female individuals.

## 2. Materials and Methods

### 2.1. Participants

Twenty-four resistance-trained participants (12 females, 12 males; age 22 ± 3 years; height 171.6 ± 7.9 cm; body mass, 69.2 ± 15.3 kg) took part in the study. Participants reported 3.4 ± 1.4 years of resistance-training experience (females 3.2 ± 1.5; males 3.6 ± 1.3 years). Relative to body mass, bench-press one-repetition maximum (1RM) was 0.99 ± 0.32 arbitrary units (a.u.) (females 0.97 ± 0.18 a.u.; males 1.01 ± 0.31 a.u.), and back squat 1RM was 1.79 ± 0.52 a.u. (females 1.73 ± 0.53 a.u.; males 1.83 ± 0.53 a.u.). Participants’ habitual sleep duration was 7.99 ± 0.12 h, in females (8.00 ± 0.11 h) and males (7.98 ± 0.13 h) monitored using the actigraphy (Kronowise 3.0 ambulatory circadian monitoring device (Kronohealth SL, Spain) during seven days.

The inclusion/exclusion criteria were as follows: (a) participants aged between 18 and 35 years; (b) absence of neuromuscular, musculoskeletal, neurological, immunological, cardio-metabolic conditions or sleep disorders; (c) the ability to perform bench press and back squat exercises; (d) no use of medications (e.g., sleep medication), drugs, stimulants, or other sports supplements (i.e., creatine, caffeine, sodium bicarbonate or melatotin) during the study period.

The menstrual cycle of each female participant was monitored using the Menstrual Distress Questionnaire (MEDI-Q) [26]. The study spanned one full menstrual cycle per participant (4 weeks), with testing sessions conducted every 7 days, ensuring that all participants were assessed across the menstrual cycle. Four participants began testing in the follicular phase, three during menstruation, and five during the luteal phase. To limit potential hormonal confounding, sleep conditions were counterbalanced across testing sessions so that experimental conditions were evenly distributed throughout the menstrual cycle.

Before enrollment in the study, participants were informed about all procedures, potential risks and discomforts related to the experiments. They provided written informed consent. The study’s design and protocol were conducted in accordance with the principles outlined in the Declaration of Helsinki and were approved by the University Ethical Investigation Committee (CEIP/2023/6/119), and registered with ClinicalTrials.gov (NCT06606626).

### 2.2. Experimental Design

A randomized, counterbalanced, cross-over trial design was employed. After a familiarization session, the protocol began with a baseline control night consisting of 8 h of sleep at home, followed by the three experimental conditions in the laboratory: (a) 8 h of sleep (NS), (b) 4 h of early sleep deprivation (ESD, 4 h of time in bed from 03:00 to 07:00 A.M., and (c) 0 h of sleep or total sleep deprivation (SD, 0 h of time in bed). Therefore, participants went to the laboratory on five separate occasions. Volunteers underwent preliminary assessments and a familiarization session during visit one. During visit two, participants performed a baseline control night and then, during visits three to five, the three experimental trials were performed in random order and separated by at least 7 days to allow complete recovery. Trials were scheduled on the same day of the week to replicate their weekly and daily habits.

Randomization was performed using a computer-generated block randomization (www.randomized.org), stratified by sex and balanced across all experimental sequences to avoid potential order effects. An external researcher, who was not involved in data collection or analysis, managed the allocation process and maintained the blinding code. This researcher revealed the assigned condition only to the participant and to the team member responsible for supervising the sleep protocol during each trial night. The researchers responsible for collecting performance and physiological data, as well as those conducting data analysis, remained blinded to the sleep condition until all analyses were completed.

In all experimental conditions, participants ingested the same dinner (542 ± 112 kcal; 0.44 ± 0.21 g/kg of protein, 0.89 ± 0.32 g/kg of carbohydrate, 0.36 ± 0.11 g/kg of fat) and then spent the night at home (baseline control night) or in the laboratory (NS, ESD or SD). In all cases, participants wore the wrist-worn actigraphy device Kronowise 3.0 (Kronohealth SL, Murcia, Spain) on their non-dominant hand, and a research team member ensured that the assigned experimental conditions were followed. Actigraphy data were processed using proprietary software and manually verified by visual inspection of activity and light exposure patterns. During the awake time at night, participants were allowed to engage in sedentary activities (e.g., playing board games, talking, watching series or movies) under the supervision of a research team member. Finally, to ensure standardization of the measurements, all tests were completed in the same laboratory using the same testing equipment, handled by the same researchers and under the same air temperature (24 ± 1 °C), humidity (32 ± 3%) and lighting (1800 lux).

### 2.3. Experimental Procedure

#### 2.3.1. Familiarization and Preliminary Assessments

On visit one, participants performed a familiarization session. During this session, body composition was measured using electric bioimpedance (Tanita BC-418, Tanita Europe B.V., Amsterdam, The Netherlands). Dietary habits were assessed through a 24 h dietary recall using the Spanish Food Composition Database (BEDCA) and CESNID Food Composition tables; while physical activity habits were evaluated using the International Physical Activity Questionnaire (IPAQ). Participants were instructed to replicate the diet from the last 24 h of each laboratory visit on the experimental trial (1634 ± 407 kcals, 1.24 ± 0.41 g/kg protein, 2.51 ± 1.08 g/kg carbohydrate, 1.05 ± 0.44 g/kg fat), and to avoid stimulants, supplements (e.g., melatonin or creatine), alcohol, and strenuous exercise for 24 h. Additionally, participants were instructed to maintain their habitual sleep patterns and avoid naps before the familiarization session or the experimental trial.

During the familiarization session, 1RM for bench press and back squat exercises was performed using a Smith machine (Multipower, Technogym, Madrid, Spain) to calculate the individualized load percentages used in the trials. The procedure began with a 10 or 20 kg load, with increments of 10 or 20 kg until the mean velocity (V_mean_) reached 0.2 m/s for the bench press and 0.4 m/s for the back squat, measured with a linear transducer (Encoder, Chronojump, Boscosystem, Barcelona, Spain). These velocity thresholds were selected because they corresponded to loads near 1RM, allowing the precise and safe estimation of maximal strength with minimal risk of over- or underestimating performance [27]. After reaching these velocity thresholds, further small increments (≤5 kg) were added to determine the 1RM. Participants performed successive maximal attempts with progressively increasing loads until failure, ensuring that the final successful attempt represented their true one-repetition maximum (1RM).

All repetitions were completed using standardized lifting techniques. The back squat was performed to a half-squat depth, with the descent reaching approximately 90° of knee flexion, confirmed by an investigator using individualized safety pins set to the appropriate height. The bench press was executed through a full range of motion, from full elbow extension to a controlled bar contact with the chest, with movement depth monitored and safety pins adjusted to maintain consistency. For both exercises, a uniform tempo was applied: a controlled eccentric phase, a 2 s isometric pause, and a concentric phase performed with maximal intended velocity. This same movement cadence was also applied during strength testing across varying loads and in the endurance assessments described below.

Then, participants underwent a familiarization test, replicating the same protocol as the experimental trials.

#### 2.3.2. Baseline Control Night and Experimental Trials

The evening before the trial, participants arrived at the laboratory at 8:30 P.M. and after the experimental condition was revealed for the next day’s trial, they ate the same dinner at the laboratory (between 9:30 and 10:00 P.M.). Then, in the baseline control night, participants returned home and slept from 11:00 P.M. to 7:00 A.M. In the NS condition, participants remained in the laboratory and slept from 11:00 P.M. to 7:00 A.M. In the ESD condition, participants slept in the laboratory from 3:00 to 7:00 A.M. (early sleep deprivation); and in the SD condition, no sleep was allowed. Early sleep deprivation (ESD, 03:00–07:00 sleep period) was chosen to emulate delayed sleep onset commonly experienced by athletes due to late training or competition schedules. Sleep was monitored using actigraphy (Kronowise 3.0 ambulatory circadian monitoring device, Kronohealth SL, Spain). Total sleep time, wake after sleep onset, sleep latency, and sleep efficiency were extracted for all conditions. During the SD condition, participants were continuously supervised by a researcher, who immediately interrupted any signs of drowsiness, and actigraphy data were reviewed to confirm that no sleep occurred.

The Kronowise 3.0 records triaxial acceleration, wrist position, temperature, and light exposure and saves data in 30 s epochs for offline analysis [28]. Raw recordings were processed with the manufacturer’s software (Kronoware 10.0/Kronowizard platform) using validated algorithms that combine activity, position, and temperature to derive total sleep time, wake after sleep onset, sleep latency, and sleep efficiency; all outputs were visually inspected to eliminate artifacts and to verify sleep/wake classification, ensuring zero sleep during the experimental condition.

**Figure 1:**
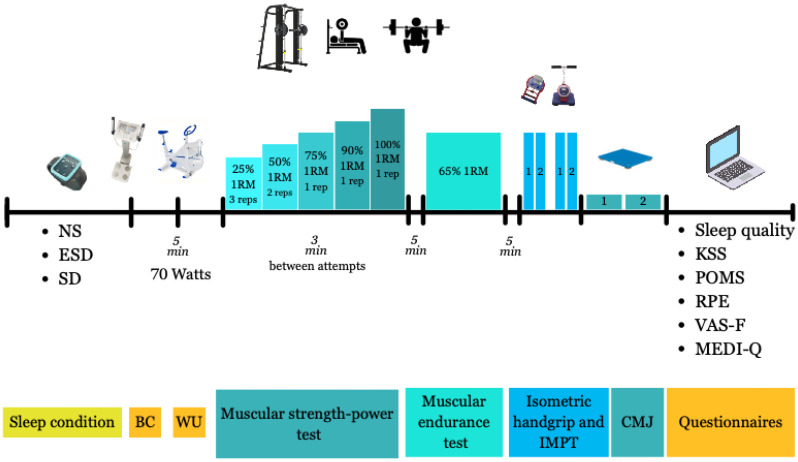
The experimental procedure of the trials. Abbreviations: BC, body composition; WU, warm-up; IMPT, Isometric Mid-Thigh Pull; CMJ, countermovement jump; NS: 8 h of sleep trial; ESD: 4 h of sleep trial; SD, 0 h of sleep trial; W, Watts; 1RM, one repetition-maximum; KSS, Karolinska Sleep Scale; POMS, Profile Of Mood States, RPE, Rating of Perceived Exertion; VAS-F, visual analog scale for fatigue. Note: the circle numbers indicate rest durations.

The experimental procedure of the trials is illustrated in Figure 1 and explained below. All experimental trials were initiated in a fasted state at the same clock time (08:30 A.M. ± 10 min) to control for circadian influences on neuromuscular performance. Hydration status was assessed at the beginning of each trial using bioelectrical impedance analysis to ensure a normal hydration state. After a 5 min standardized warm-up on a stationary cycloergometer, the strength tests were conducted. Mean velocity (V_mean_), calculated as the average barbell velocity across the entire concentric phase of the lift, including both propulsive and braking phases, were recorded at five increasing loads: 25%, 50%, 75%, 90%, and 100% 1RM for bench press and back squat exercises using a linear encoder (Encoder, Chronojump, Boscosystem, Barcelona, Spain) attached to the bar on a Smith machine. Participants performed three repetitions at 25% 1RM, two repetitions at 50% 1RM, and one repetition for 75%, 90%, and 100% 1RM. A three-minute passive recovery period was provided between sets. These multiple relative loads (25–100% 1RM) were tested to capture the full force–velocity profile, allowing the assessment of load-specific effects on strength while using repetition schemes appropriate to each intensity for safety and ecological validity.

After five minutes of recovery, muscular endurance was evaluated by performing one set at 65% 1RM for both bench press and back squat exercises, continuing until task failure. Participants followed the same technique instructions for each repetition, as mentioned before. A five-minute passive recovery separated each set and exercise, during which participants remained seated and relaxed without engaging in any physical activity, using electronic devices, or conversing. The number of repetitions, V_mean_, the mean velocity of the fastest repetition of the set (V_fastest_), and the mean velocity of the last repetition of the set (V_last_) were recorded. For, V_mean_ the lowest number of repetitions performed in any experimental condition was used to calculate the average velocity produced. This ensures that V_mean_ reflects performance over an equivalent number of repetitions across all conditions, allowing comparisons between experimental trials.

After five minutes, isometric handgrip and isometric mid-thigh pull (IMTP) strength tests were performed (Grip-D, Takei Scientific Instruments Co., Ltd., Tokyo, Japan). Two repetitions were completed for each test, with an additional repetition if differences exceeded 10%. Each repetition involved 5 s of maximal contraction with 30 s of passive recovery. Countermovement jump performance (CMJ, without arm swing) was then assessed on a force platform (Kistler 9229a, Winterthur, Switzerland). Participants completed two attempts with 1 min of passive recovery, with an additional attempt if differences exceeded 10%. For all tests, the best performance was recorded.

Reliability of these measurements was assessed using data from the familiarization and the control night of 8 h of sleep at home. The coefficient of variation (CV) was calculated as the typical error divided by the pooled mean and expressed as a percentage. In the strength performance against different loads, the CV for bench press V_mean_ was <4.4% and <4.9% for back squat. In the muscular endurance test, the CV for bench press and back squat V_mean_ was 6.7% and 7.2%, respectively. The CV isometric handgrip and IMPT were 3.4% and 4.1%, whereas vertical jump height and power were 4.2% and 5.8%, respectively.

Finally, participants completed questionnaires about their perception of sleep, mood and effort. Participants rated their sleep quality using a scale from 1 (very poor) to 5 (very good) and also completed the Karolinska Sleepiness Scale, which uses a 1-to-10-point scale. The Profile of Mood States (POMS) was used to assess participants’ mood. This questionnaire consisted of 29 mood-related items, which participants rated from 0 (nothing) to 4 (extremely) according to their feelings at that moment, divided into six scales: tension, depression, anger, vigor, fatigue and confusion. Finally, they were asked about their perceived effort (RPE) and visual analog scale for fatigue (VAS-F) before and after the trial.

### 2.4. Statistical Analysis

The sample size calculation revealed that twenty-four participants (12 females and 12 males) were sufficient for detecting differences in muscular strength/power and endurance tests with an effect size of 0.3 (α = 0.05; 1−β = 0.80) (v3.1, G*power, Dusseldorf University, Dusseldorf, Germany), based on a previous meta-analysis reporting a small-to-moderate effect of sleep deprivation on muscular performance [20].

Data was analyzed using the statistical package SPSS v29.0 (SPSS Inc., Chicago, IL, USA) and GraphPad Prism (v8, GraphPad Software Inc., La Jolla, CA, USA). Shapiro-Wilks was used to test the normality of the data (*p* > 0.05). After normal distribution confirmation, strength, power and endurance performance data were analyzed using an ANCOVA with sleep condition (8 h vs. 4 h vs. 0 h) as a within-subject factor, sex (female vs. male) as between-subjects factors, and the baseline control night (8 h of sleep at home) as covariate. Baseline control night data were included as covariates to account for potential effects of sleeping outside the habitual environment and to more accurately isolate the impact of laboratory-based partial and total sleep deprivation on performance outcomes. Trial order was randomized and balanced across participants; therefore, it was not included as a fixed factor in the ANCOVA model. The within-subject design, together with inclusion of baseline control night data as a covariate, ensures that potential sequence or period effects are minimized. Homoscedasticity assumptions were assessed using Levene’s tests, sphericity was verified with Mauchly’s test and, if violated, the Greenhouse-Geisser correction was applied. Post hoc comparisons were adjusted using the Holm-Bonferroni method.

Values are reported as mean ± standard deviation (SD). The significance level was set at *p* < 0.05. Effect sizes for the ANCOVA were expressed as partial eta squared (η_p_^2^) and interpreted as small (0.01–0.06), medium (0.06–0.14), and large (>0.14). For pairwise or post hoc comparisons, effect sizes were expressed as Hedges’s *g* and interpreted as trivial (0.00–0.19), small (0.20–0.49), medium (0.50–0.79), and large (≥0.80).

## 3. Results

### 3.1. Strength Performance Against Different Loads

**Figure 2:**
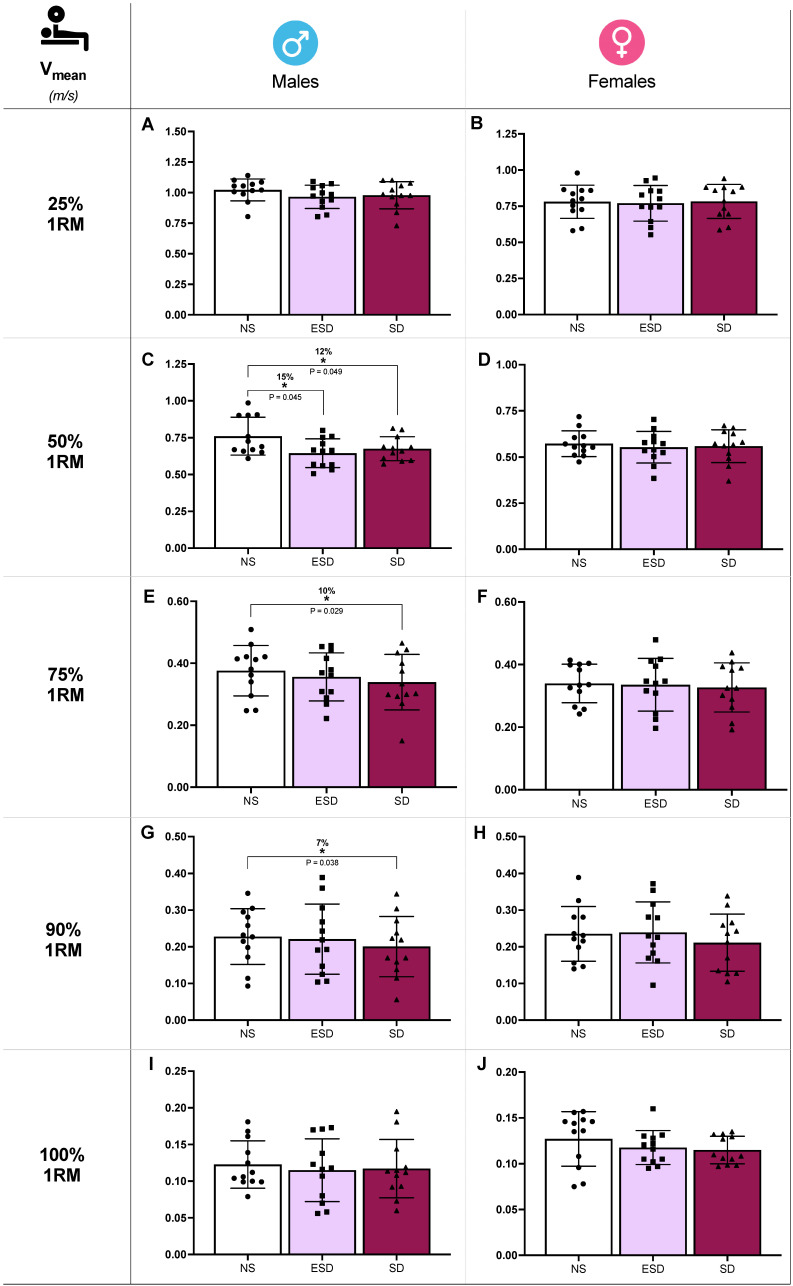
Strength performance against different loads in bench press across sleep conditions and sex. Mean velocity (V_mean_) at 25% 1RM in males (**A**) and females (**B**), at 50% 1RM in males (**C**) and females (**D**), at 75% 1RM in males (**E**) and females (**F**), at 90% 1RM in males (**G**) and females (**H**), at 100% 1RM in males (**I**) and females (**J**). Abbreviations: NS, 8 h of sleep; ESD, 4 h of early sleep deprivation; SD, 0 h of sleep or total sleep deprivation. * *p* < 0.05 in comparison to NS.

Differences in bench press mean velocity (V_mean_) across sleep conditions in male and female participants are illustrated in Figure 2. At 50% 1RM, sleep and sleep by sex effects were found in V_mean_ (*p* = 0.022 and 0.045, η_p_^2^ = 0.12 and 0.09). Partial comparison revealed a statistically significant decrease in V_mean_ of male participants when NS was compared to ESD (15%, 0.760 ± 0.128 vs. 0.645 ± 0.097 m/s; *p* = 0.045, *g* = 0.66) and to SD (12%, 0.760 ± 0.128 vs. 0.667 ± 0.081 m/s; *p* = 0.049, *g* = 0.50). These differences were not found in female participants when NS was compared to ESD (3%, 0.573 ± 0.069 vs. 0.553 ± 0.086 m/s; *p* = 0.298, *g* = 0.15) or to SD (2%, 0.573 ± 0.069 vs. 0.559 ± 0.089 m/s; *p* = 0.593, *g* = 0.17).

Moreover, despite no sleep or sleep by sex effect found in the ANCOVA model, V_mean_ showed a decrease in SD compared to NS at 75% 1RM (10%, 0.376 ± 0.077 vs. 0.339 ± 0.089 m/s; *p* = 0.029, *g* = 0.53; Figure 2E) and 90% 1RM (7%, 0.228 ± 0.079 vs. 0.200 ± 0.082 m/s; *p* = 0.038, *g* = 0.48; Figure 2G). These comparisons at 75% and 90% 1RM are presented for descriptive purposes only and should not be interpreted as confirmatory evidence.

**Figure 3:**
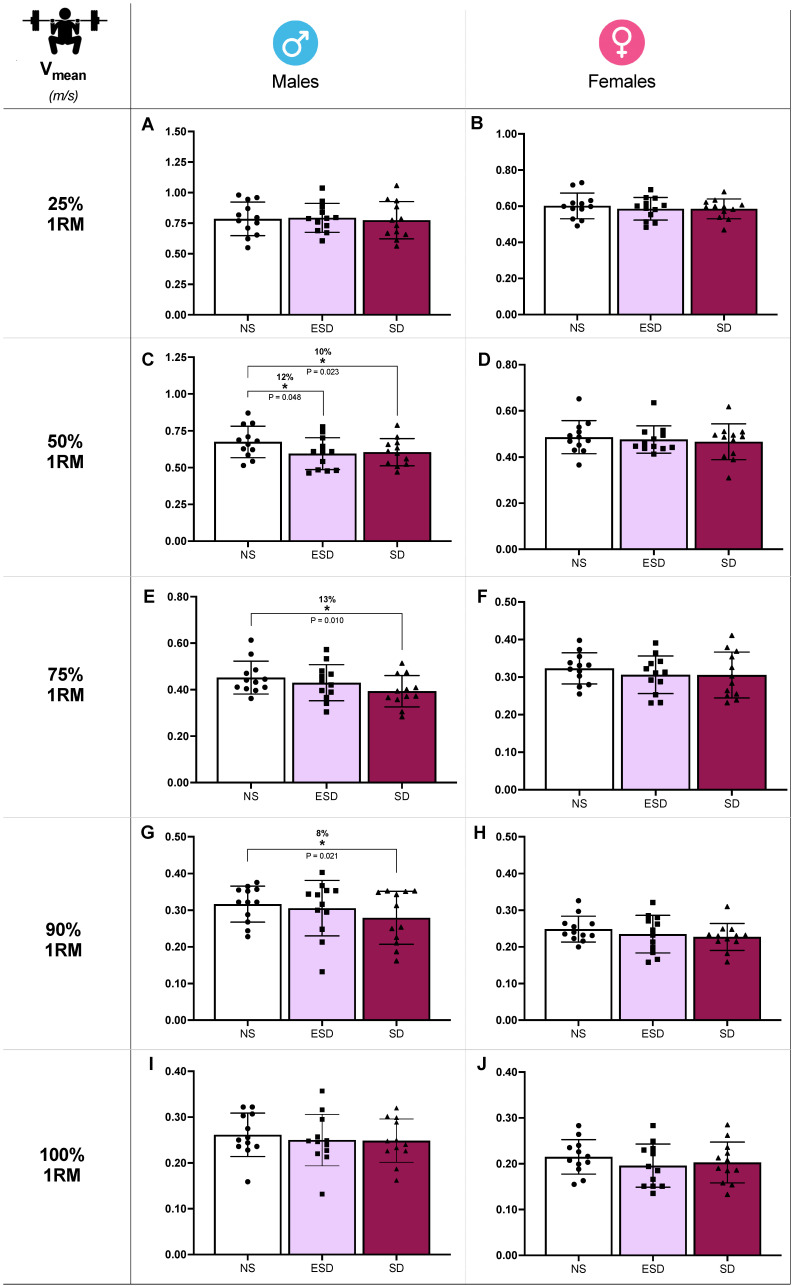
Strength performance against different loads in back squat across sleep conditions and sex. Mean velocity (V_mean_) at 25% 1RM in males (**A**) and females (**B**), at 50% 1RM in males (**C**) and females (**D**), at 75% 1RM in males (**E**) and females (**F**), at 90% 1RM in males (**G**) and females (**H**), at 100% 1RM in males (**I**) and females (**J**). Abbreviations: NS, 8 h of sleep; ESD, 4 h of early sleep deprivation; SD, 0 h of sleep or total sleep deprivation. * *p* < 0.05 in comparison to NS.

Differences in back squat mean velocity (V_mean_) across sleep conditions in male and female participants are illustrated in Figure 3. At 50% 1RM, sleep and sleep by sex effects were found in V_mean_ (*p* = 0.032 and 0.046, η_p_^2^ = 0.12 and 0.09). Partial comparison revealed a statistically significant decrease in V_mean_ of male participants when NS was compared to ESD (12%, 0.675 ± 0.107 vs. 0.595 ± 0.108 m/s; *p* = 0.048, *g* = 0.86) and to SD (10%, 0.675 ± 0.107 vs. 0.605 ± 0.093 m/s; *p* = 0.023, *g* = 0.81). These differences were not found in female participants when NS was compared to ESD (2%, 0.486 ± 0.072 vs. 0.476 ± 0.059 m/s; *p* = 0.515, *g* = 0.17) or to SD (4%, 0.486 ± 0.072 vs. 0.466 ± 0.077 m/s; *p* = 0.347, *g* = 0.16).

Moreover, despite no sleep or sleep by sex effect found in the ANCOVA model, V_mean_ showed a decrease in SD compared to NS at 75% 1RM (13%, 0.452 ± 0.070 vs. 0.393 ± 0.067 m/s; *p* = 0.010, *g* = 0.53; Figure 3E) and 90% 1RM (8%, 0.317 ± 0.049 vs. 0.279 ± 0.072 m/s; *p* = 0.021, *g* = 0.43; Figure 3G). These comparisons at 75% and 90% 1RM are presented for descriptive purposes only and should not be interpreted as confirmatory evidence.

### 3.2. Muscular Endurance Test

**Figure 4:**
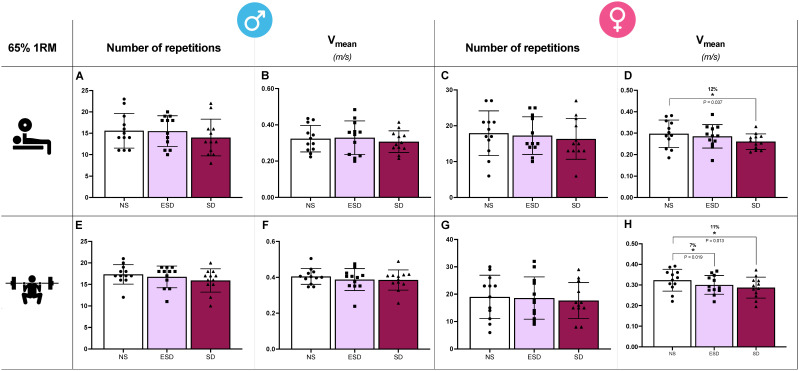
Muscular endurance tests in bench press and back squat across sleep conditions and sex. In the bench press exercise, the number of repetitions and mean velocity (V_mean_) in males (**A**,**B**) and females (**C**,**D**). In the back squat exercise, the number of repetitions and mean velocity (V_mean_) in males (**E**,**F**) and females (**G**,**H**). Abbreviations: NS, 8 h of sleep; ESD, 4 h of early sleep deprivation; SD, 0 h of sleep or total sleep deprivation. * *p* < 0.05 in comparison to NS.

Differences in the number of repetitions and V_mean_ across sleep conditions in male and female participants for the bench press and back squat exercises are illustrated in Figure 4. In back squat exercise, sleep and sleep by sex effect were found in V_mean_ (*p* = 0.001 and 0.047, η_p_^2^ = 0.21 and 0.10). Partial comparison revealed that these effects were found in female participants. In V_mean_, a decrease was observed when comparing NS to ESD (7%, 0.323 ± 0.053 vs. 0.300 ± 0.048 m/s; *p* = 0.013, *g* = 0.59; Figure 4H) and to SD (11%, 0.323 ± 0.053 vs. 0.287 ± 0.051 m/s; *p* = 0.018, *g* = 0.048; Figure 4H). These differences were not found in male participants when NS was compared to ESD (4%, 0.405 ± 0.044 vs. 0.387 ± 0.61 m/s; *p* = 0.166, *g* = 0.21) or to SD (5%, 0.405 ± 0.044 vs. 0.385 ± 0.057 m/s; *p* = 0.191, *g* = 0.24).

Moreover, despite no sleep or sleep by sex effect found in the ANCOVA model, V_mean_ showed a decrease in SD compared to NS in females bench press (12%, 0.297 ± 0.064 vs. 0.260 ± 0.036 m/s; *p* = 0.037, *g* = 0.41; Figure 4D). These differences were not observed in male participants, and no other sleep or sleep by sex condition differences were observed across any other variable (V_last_ and V_fastest_).

### 3.3. Isometric Strength and Vertical Jump

No sleep or sleep by sex differences were found in isometric handgrip strength of the dominant hand (*p* = 0.181, η_p_^2^ = 0.072 and *p* =0.428, η_p_^2^ = 0.04), non-dominant hand (*p* = 0.229, η_p_^2^ = 0.064 and *p* = 0.803, η_p_^2^ = 0.013) or IMPT (*p* = 0.677, η_p_^2^ = 0.023 and *p* = 0.338, η_p_^2^ = 0.049). Similarly, in the vertical jump test, no sleep or sleep by sex differences were noted in height (*p* = 0.832, η_p_^2^ = 0.013 and *p* = 0.964, η_p_^2^ = 0.004) or power output (*p* = 0.603, η_p_^2^ = 0.026 and *p* = 0.500, η_p_^2^ = 0.034).

### 3.4. Sleep and Questionnaires About Mood and Perception of Effort

Total sleep time in the baseline control night at home was 7.72 ± 0.28 h in males and 7.74 ± 0.33 h in females, in the NS it was 7.63 ± 0.27 h in males and 7.60 ± 0.31 h in females and in the ESD it was 3.74 ± 0.24 h in males and 3.70 ± 0.28 h in females. Sleep monitoring revealed significant differences in sleep latency (*p* = 0.033, η_p_^2^ = 0.152). Differences were found between NS and ESD (26.7 ± 19.3 vs. 14.5 ± 11.0 min; *p* = 0.014, *g* = 0.75) and effect was observed in females (23.3 ± 16.6 vs. 10.9 ± 6.3 min; *p* = 0.020, *g* = 0.92) but not in males (30.1 ± 22.0 vs. 18.0 ± 13.5 min; *p* = 0.170, *g* = 0.61). Moreover, sleep efficiency did not differ significantly (*p* = 0.227, η_p_^2^ = 0.065), reporting similar values between NS and ESD (93.1 ± 6.0 vs. 94.2 ± 5.0%; *p* = 0.533; *g* = 0.18), in either males (90.7 ± 7.1 vs. 92.2 ± 6.0%; *p* = 0.635, *g* = 0.22) or females (95.6 ± 3.4 vs. 96.1 ± 2.7%; *p* = 0.652, *g* = 0.17). No differences were found in wake after sleep onset (*p* = 0.456, η_p_^2^ = 0.052), reporting similar values in baseline control night at home in males 0.50 ± 0.25 min and females 1.58 ± 0.58 min (*p* = 0.456, *g* = 0.34), in NS in males 2.00 ± 1.01 min and 2.73 ± 0.20 min in females (*p* = 0.242, *g* = 0.20) and in ESD 0.58 ± 0.20 min in males and 0.43 ± 0.12 in females (*p* = 0.148, *g* = 0.22).

In the Karolinska Sleepiness Scale, a sleep effect was found (*p* < 0.002, η_p_^2^ = 0.38) and the differences were observed in both sexes in SD where KSS was higher after the trial compared to NS (33%, *p* = 0.001, *g* = 1.17) and ESD (15%, *p* = 0.017, *g* = 0.55) as expected. Moreover, in the subjective quality of sleep, a sleep by sex effect was detected (*p* = 0.022, η_p_^2^ = 0.162), revealing that in female participants, quality of sleep was lower in ESD compared to NS (15%, *p* = 0.032, *g* = 0.84). An effect that was not found in male participants (~5%, *p* > 0.110, *g* > 0.015).

Finally, in RPE, a sleep effect was found (*p* < 0.001, η_p_^2^ = 0.305). This difference was observed in both sexes in SD where RPE was higher after the trial compared to NS (20.6%, *p* < 0.001, *g* = 3.49) and ESD (16%, *p* = 0.014, *g* = 3.15). Finally, no statistically significant differences were found in the VAS-F (*p* > 0.342) or the six mood state scales (*p* > 0.210).

## 4. Discussion

This study evaluated sex differences in the effects of total (0 h) and partial (4 h) sleep deprivation on strength, power, and endurance performance during bench press and back squat in resistance-trained participants. To our knowledge, no previous study has examined sex-specific impairments from sleep deprivation in strength, where women remain underrepresented, and evidence on dynamic contractions is limited. Results showed that total and partial sleep deprivation affect males and females differently. In males, strength declined, with reduced mean velocity during bench press and back squat at 50% 1RM. In females, endurance was impaired at 65% 1RM, as evidenced by lower mean velocity, particularly in the back squat. These findings indicate that sleep deprivation produces sex-related response patterns at moderate loads, reducing strength in males and endurance in females.

Strength performance across different loads (25–100% 1RM) revealed that total and partial sleep deprivation caused a 7–13% decrease in the ability to generate velocity (V_mean_), particularly at moderate loads (50% 1RM) in bench press and back squat exercises, in male but not in female participants. A meta-analysis reported a small overall reduction in strength following sleep deprivation (~2.9%) [20]; however, effects across individual studies have been inconsistent. Some investigations report significant impairments [5,29,30], whereas many others show no change in maximal strength across a range of muscle groups and populations [3,4,29,31,32,33]. Notably, null findings have been reported for knee extensors and flexors and elbow flexors in healthy [3,4,29,31,32,33,34,35], physically active [32,33], trained [29], and athletic populations [31], as well as in sedentary eumenorrheic females [3]. These non-significant effects have been observed across different testing times (morning [3,4,29,31,32] and evening [3]) and following both total and partial sleep deprivation protocols [3,4] of varying durations (1–2.5 days [29,31,33,35]). However, in contrast, Souissi et al. [29] found in twelve judokas that late but not early sleep deprivation caused a reduction in MVC of elbow flexors in the afternoon (4:00 P.M.) but not in the morning (9:00 A.M.). Additionally, using dynamic contractions, Reilly and Piercy [5] analyzed the effect of 3 h sleep (from 02:45 to 5:45 A.M.) for three successive nights on the maximal load able to mobilize in bicep curl, bench press, leg press and deadlift performed between 5:00 and 7:00 P.M. after each day of sleep deprivation. A decline in performance was found in all exercises, particularly after the second day of sleep deprivation and onwards. Beyond the interaction between time awake and time of day on the effects of sleep loss on neuromuscular performance [36,37], the available evidence, together with the findings of the present study, suggests that acute sleep deprivation does not substantially impair performance in tasks requiring either maximal force production (e.g., >90% 1RM, isometric handgrip, or maximal voluntary contractions) or maximal movement velocity (e.g., ≤25% 1RM or countermovement jumps) when assessed in the morning after sleep loss. In contrast, when tasks require a moderate combination of force, velocity, and neural control, such as lifting at moderate loads (~50% 1RM), the detrimental effects of both partial and total sleep deprivation on strength and power become more apparent, even during morning testing and in both upper-body (bench press) and lower-body (back squat) exercises. These findings may suggest that sleep deprivation may primarily disrupt neural efficiency and intermuscular coordination [38], rather than directly impairing motor neuron excitability or maximal force output, which appear to decline mainly with prolonged wakefulness [36,38].

Interestingly, at higher loads (75% and 90% 1RM), mean velocity also showed reductions under total but not early partial sleep deprivation in both bench press and back squat. Although these effects were exploratory, they suggest that sleep loss may potentially reduce neuromuscular efficiency even at heavier loads, reflecting early signs of central or peripheral fatigue.

In the muscular endurance test, total and partial sleep deprivation resulted in a 7–12% decrease in the ability to generate velocity in female participants. Similar evidence can be found in the literature [32,39]. Previous evidences show that total sleep deprivation does not impair knee flexion-extension performance at 70% of isometric peak torque in physically active males, as long as maximal effort is sustained [32]. In contrast for dynamic contractions, Cook, Beaven, Kilduff and Drawer [39] reported a decrease in total volume or bench press, squat and bent row exercises after performing four sets at 85% 1RM until exhaustion under partial sleep deprivation (≤6 h of self-reported sleep at home). However, this decrease in muscular endurance only seems to occur when the task is performed until exhaustion [40]. Hence, although fatigue and sleep deprivation are closely connected [41], some findings suggest that central fatigue and consequent changes in supraspinal input to the motoneuron pool are not exacerbated after sleep disturbances [38,42], which may suggest that peripheral factors are responsible for the decrease in muscular endurance observed after total and partial sleep deprivation. However, central mechanism cannot be avoided since this effect seems to occur in tasks with high neuromuscular demand (i.e., back squat vs. bench press).

Regarding sex-based analyses, the present study found that both total and partial sleep deprivation reduced strength performance in male participants, whereas muscular endurance performance was primarily impaired in female participants. Unfortunately, only one previous study has analyzed the effect of sleep deprivation on strength, power or endurance performance in female participants [3]. In eight sedentary eumenorrheic females, Bambaeichi, Reilly, Cable and Giacomoni [3] measured knee extensors and flexors of the dominant leg at 6:00 A.M. and 6:00 P.M. after 8 h sleep and 2.5 h of early sleep deprivation, not reporting differences in muscular strength caused by sleep deprivation at any time point. Prior evidence suggests that males generally exhibit a larger relative area of type II muscle fibers and smaller proportional areas of type I fibers compared to females [43,44]. This difference may be associated with faster contractile properties and greater reliance on glycolytic metabolism in males [45,46], whereas females typically display higher oxidative capacity in whole muscle tissue [45,47]. Such distinctions could potentially explain at least in part why sleep deprivation might affect males more on tasks requiring high force and power output (e.g., ~50% 1RM). Conversely, females often demonstrate greater fatigue resistance during sustained or repetitive contractions, potentially due to differences in muscle metabolism and relaxation dynamics [48,49]. Additionally, differences in neuromuscular recruitment strategies may also play a role. Some studies have reported higher motor unit firing rates in females than in males, particularly at higher loads [50,51]. In tasks performed until failure, such as the endurance tests used in this study, a progressive increase in motor unit recruitment and firing rate is required. Therefore, it is conceivable that sleep deprivation could differentially disrupt this process between sexes. However, while these physiological factors could plausibly contribute to the sex-specific patterns observed, these mechanisms were not directly measured in the present study and should therefore be interpreted cautiously.

Finally, questionnaire outcomes support the main findings, as RPE was higher under sleep deprivation in both sexes, reflecting greater perceived effort that aligns with the observed decreases in mean velocity [22]. No changes in fatigue or mood were observed, suggesting that the performance impairments were specifically related to effort perception rather than general affective state.

## 5. Limitations

Several limitations should be acknowledged. First, although sex-specific performance differences were observed, the underlying mechanisms remain speculative and should be interpreted as hypotheses rather than confirmed explanations. Future studies incorporating direct physiological and neurophysiological measures (e.g., cerebral and muscle oxygenation, electromyography, and hormonal responses) are needed to clarify the neuromuscular and metabolic factors underlying these findings. Additionally, all testing was conducted at a single morning time point (8:30 A.M.), limiting insight into how performance impairments may vary across the day. This is particularly relevant given evidence that acute sleep restriction reduces strength, power, and endurance performance in both athletic and non-athletic populations and that these decrements are influenced by circadian timing, with larger impairments typically occurring following late-night or total sleep deprivation during afternoon or evening sessions [20,21,22,52,53]. Therefore, although performance decrements were observed in the present study, they may have been underestimated due to the exclusive use of morning assessments.

## 6. Conclusions

In conclusion, sleep deprivation induces a sex-related response pattern of impairment in resistance-trained individuals at moderate loads (50–65% 1RM). In males, both total and partial sleep deprivation significantly reduced mean velocity at 50% 1RM in both bench press and back squat exercises. In females, sleep deprivation primarily affected muscular endurance (65% 1RM), with reductions in mean velocity, particularly during back squat exercise. These results highlight the importance of considering sex-specific responses when evaluating the impact of sleep deprivation on athletic performance.

## Data Availability

The original contributions presented in the study are included in the article; further inquiries can be directed to the corresponding author.

## Abbreviations

The following abbreviations are used in this manuscript:

1RM	One-repetition maximum
AM	*Ante meridiem*
a.u.	Arbitrary units
BEDCA	Spanish Food Composition Database
CMJ	Counter movement jump
ESD	4 h of early sleep deprivation trial
IPAQ	International Physical Activity Questionnaire
IMTP	Isometric mid-thigh pull
MEDI-Q	Menstrual Distress Questionnaire
NS	8 h of sleep trial
PM	*Post meridiem*
POMS	Profile of Mood States questionnaire
RPE	Rate of perceived exertion
SD	0 h of sleep trial
V_mean_	Mean velocity
